# Modulation of Cilia Motility by Vortex-Ultrasound-Induced Shear Stress

**DOI:** 10.1021/acsnano.6c06231

**Published:** 2026-07-15

**Authors:** Thi-Nhan Phan, Hsien-Chu Wang, Ching-Hsiang Fan, Chung-Han Huang, Yin Fang, Yucheng Luo, Zhichao Ma, I-Hsuan Lin, Won-Jing Wang, Yu-Chun Lin, Chih-Kuang Yeh

**Affiliations:** † Department of Biomedical Engineering and Environmental Sciences, 34881 National Tsing Hua University, Hsinchu 30013, Taiwan; ‡ Institute of Molecular Medicine, National Tsing Hua University, Hsinchu 30013, Taiwan; § Department of Biomedical Engineering, National Cheng Kung University, Tainan 701401, Taiwan; ∥ Center of Transformative Bioelectronic Medicine, College of Medicine, National Cheng Kung University, Tainan City 701, Taiwan; ⊥ Medical Device Innovation Center, National Cheng Kung University, Tainan 701401, Taiwan; # Institute of Medical Robotics, School of Biomedical Engineering, 12474 Shanghai Jiao Tong University, Shanghai 200030, China; ¶ Institute of Biochemistry and Molecular Biology, 34914 National Yang Ming Chiao Tung University, Taipei 300093, Taiwan; ∇ Department of Medical Science, National Tsing Hua University, Hsinchu 30013, Taiwan; ○ Department of Biomedical Sciences and Engineering, Tzu Chi University, Hualien 970374, Taiwan; ⧫ Department of Biomedical Engineering, Chung Yuan Christian University, Taoyuan 320314, Taiwan

**Keywords:** cilia motility, vortex ultrasound, shear stress, ultrasound, neuromodulation

## Abstract

The precise and noninvasive modulation of primary ciliary mechanotransduction remains a significant challenge in cell biology. Current approaches induce cilia motility—including microfluidic flow, optical tweezers, magnetic actuation, and genetic or optogenetic techniques—are constrained by low spatiotemporal precision and poor suitability for in vivo applications. Here, we present a noninvasive approach using vortex ultrasound (VUS) to generate localized shear stress via helical acoustic streaming. Using a 3.5 MHz transducer, VUS-generated shear stresses were approximately 5-fold higher than for conventional focused ultrasound, inducing cilia deflections of up to 80°. This mechanical stimulation triggered cilia-dependent calcium influx via ciliary ion channels, including transient receptor potential vanilloid 4 (TRPV4) and transient receptor potential polycystin 2 (TRPP2), demonstrating the direct activation of primary ciliary mechanotransduction by VUS-induced shear stress. These findings indicate VUS is a powerful tool for ciliary mechanobiology that can offer a scalable physical modality for investigating and manipulating cilia-associated signaling pathways in intact biological systems.

## Graphical abstract

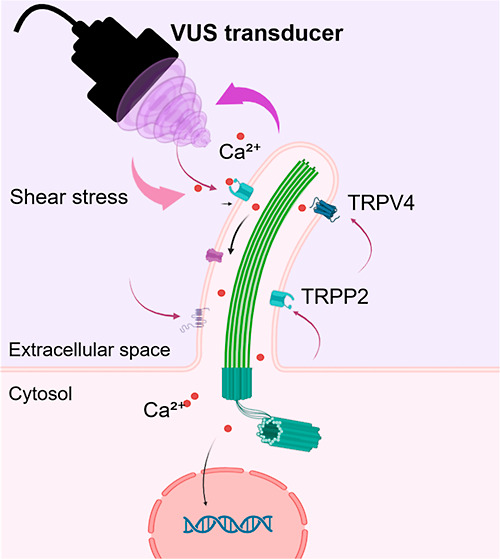

Primary cilia are specialized microtubule-based sensors, which present on the surface of most mammalian cell types.^1^ They transduce a wide range of environmental cues—inclusive of mechanical stimuli, chemical, electrical, and particularly fluid-flow-induced shear stress—into intracellular signaling pathways that modulate cellular processes such as proliferation, migration, and differentiation, and the polarity of planar cells.^2–5^ Stimulation by shear stress induces cilia deflection—a hallmark of ciliary mechanosensitivity and a defining feature of ciliary mechanotransduction^6–8^ —that triggers intracellular calcium influx and subsequent modulation of downstream gene expression. Shear-induced calcium signaling initiates transcriptional responses across diverse cell types, including vascular endothelial cells, hepatocytes, and osteocytes.^6–8^ Mechanotransducers such as polycystin 1 and polycystin 2, which are localized to the ciliary membrane, are activated by mechanical deflection of the cilium and play critical roles in calcium homeostasis and mechanotransduction-based signaling.^9,10^

Defects in cilia motility and disruptions to ciliary signaling during excitation by fluid-flow-induced shear stress represent key pathogenic mechanisms underlying ciliopathies,^11,12^ which is a group of disorders affecting multiple organ systems, including the brain, kidneys, eyes, liver, and skeleton. Ciliary dysfunction in the nervous system impairs cerebellar morphogenesis,^13,14^ compromises the establishment of adult neural stem-cell niches, and contributes to syndromic conditions such as orofaciodigital syndrome, which is characterized by central nervous system malformations, intellectual disability, ataxia, and retinal dystrophy.^15^ These diverse effects have led to recent efforts to develop therapeutic strategies for ciliopathies, increasingly focused on elucidating the mechanosensory functions of cilia in response to fluid-flow-induced shear stress, with particular emphasis on structural deflection, motility regulation, and downstream tissue-level outcomes.

Current approaches for inducing primary cilium deflection include fluid-flow-induced shear stress, optical tweezers, magnetic actuation, genetic and optogenetic manipulation, and acoustic or ultrasound-based techniques.^16–21^ Despite significant progress, the translation potential of these methods has been restricted by most of them relying on indirect mechanical stimulation and lacking sufficient spatial specificity, temporal resolution, and precise control over cilia deflection. It is therefore essential to develop a novel technique capable of producing physiologically relevant cilium deflection with high spatiotemporal precision to advance both fundamental cilia research and therapeutic interventions targeting ciliopathies.

This study investigated whether the above-described limitations could be overcome using vortex ultrasound (VUS), which is an advanced modality of conventional ultrasound. VUS generates helical acoustic wavefronts through phase dislocations along the axial axis, which induces a high-pressure annular region that surrounds a central pressure-null core. This acoustic potential well induces fluid streaming to enable the contactless, high-precision manipulation, trapping, and transport of particles and biological structures.^22,23^ VUS has recently been increasingly adopted across biomedicine, materials science, and microfluidics, with reported applications including droplet and particle manipulation for material synthesis and drug delivery,^24^ integration into continuous-flow microfluidic systems, and operation as an acoustic lens to guide and steer laser beams.

Beyond these applications, VUS has been employed for the isolation and sorting of cells based on their size, stiffness, and acoustic properties,^25,26^ for the contact-free assembly of cell spheroids and organoids through spatial organization,^27^ for improving intracellular delivery via membrane deformation induced by focused acoustic fields,^24^ and for scaffold-free tissue patterning involving multiple cell types.^28^ One notable characteristic of VUS is the generation of high flow velocity and localized shear stresses that can reach peak values up to 4-fold higher than those produced by conventional nonvortex ultrasound and comparable to the physiological shear stresses within arterial vessels.^29–31^ This unique capability has been utilized to not only disrupt thrombi^29–32^ but also modulate ciliary activity through acoustic streaming-induced shear stress. The integration of VUS with the guidance of an imaging system is a promising approach for translation into in vivo and clinical applications. Recent in vivo studies have demonstrated its capability in thrombolysis, blood-flow imaging, and targeted drug delivery. Notably, miniaturized VUS catheters have achieved effective thrombolysis in porcine pulmonary arteries under the guidance of a C-arm image system.^33^ In addition, integration of VUS with volumetric super-resolution imaging has enabled targeted microbubble accumulation and real-time monitoring in rat cerebral vessels.^34^ Besides, VUS has been applied for guiding kidney stone fragments in the pig’s bladder,^35^ real-time imaging of venous blood flow in the lower limbs of rabbits, and selective navigation of individual drug carriers through microbubble-mediated transport mechanisms.^22,36,37^

Here, we introduce VUS as a noninvasive approach for modulating neuronal cilia via tornado-like acoustic streaming that generates highly localized shear stress. This shear stress induces cilia motility in vitro and promotes calcium influx, thereby increasing neuronal activity. We hypothesized that VUS-induced shear stress activates mechanosensitive ion channels located on the ciliary membrane, which in turn trigger downstream signaling pathways. We tested this hypothesis by first quantifying the relationships among VUS streaming dynamics, acoustic parameters, and the resulting shear stress using particle image velocimetry (PIV). We then examined the effects of VUS-induced shear stress on cilia motility, as assessed by measuring cilia deflection angles, and on intracellular calcium influx. To further establish the contribution of cilia to VUS-induced calcium signaling, we selectively depleted calcium stores in either the extracellular medium or the cytosol. For comparison, shear stress generated by conventional focused ultrasound (FUS) was applied under identical experimental conditions. We next systematically varied acoustic parameters, including acoustic pressure (300–600 kPa), number of cycles (10–2000), and stimulation duration (10–30 s), to characterize their influence on ciliary dynamics and calcium signaling. Finally, to determine the role of ciliary ion channels in sensing the VUS-induced shear stress, we employed pharmacological blockers to inhibit key mechanosensitive channels expressed on neuronal cilia.

The findings of this study establish VUS as a versatile approach for precisely manipulating cilia motility and modulating neuronal activity, with potential for further studies of ciliopathies and other neurological disorders associated with ciliary dysfunction in in vitro models.

## Results

### 3.5 MHz VUS Transducer Can Induce Shear Stress

We developed a VUS system capable of generating highly localized mechanical shear stress to stimulate cilia motility. The system comprises a planar 3.5 MHz transducer (diameter = 30 mm) and a focused-vortex lens to form the VUS transducer. Acoustic fields in the *xy* and *xz* planes were measured using a hydrophone (Figure S1B) with a focal length of 50 mm and a beam width of 4 mm. The helical phase fronts of the vortex beam produced a central void, observed as a dark region at the beam center. At *z* = 50 mm in the horizontal (*xy*) plane, the surrounding high-pressure zone encircled the void in a spiral pattern (Figure 1A). The cross-section through the beam in Figure 1C further confirms that the pressure distribution formed a potential well. As a control, we used a FUS transducer (3.5 MHz, diameter = 30 mm, focal length = 50 mm). The VUS waveform also confirmed the angular momentum, as demonstrated by the corresponding phase map, which exhibits continuous phase shifts from – π to π radians (Figure 1B). To visualize VUS-induced flow patterns, 1 μm YG-labeled microspheres were suspended in degassed distilled water, exposed to VUS [acoustic pressure = 500 kPa, 2000 cycles, pulse repetition frequency (PRF) = 70 Hz], and then quantified using real-time PIV microscopy (Figure S1C). The shear stress generated by VUS was 0.37 ± 0.02 dyn/cm^2^ [(mean ± standard error of the mean (SEM)], which was 5.0-fold higher than that produced by FUS under identical acoustic parameters (acoustic pressure = 500 kPa, 2000 cycles, PRF = 70 Hz) (0.050 ± 0.008 dyn/cm^2^) while being 2.3-fold lower than that produced by FUS at the higher acoustic pressure of 1300 kPa (2000 cycles, PRF = 70 Hz) (0.69 ± 0.13 dyn/cm^2^) (*p* < 0.05) (Figure 1E). The flow vectors and velocity in Figure 1D show that the fluid streaming generated by VUS exhibits a tornado-like pattern, similar to that identified for the acoustic field (Figure 1A), whereas that generated by FUS is concentrated at the focal point of the FUS transducer. The results further showed that the shear stress peaked for stimulation by VUS at an acoustic pressure of 600 kPa (0.45 ± 0.12 dyn/cm^2^), which was significantly higher than the stresses for acoustic pressures of 300, 400, and 500 kPa: 0.05 ± 0.04, 0.15 ± 0.02, and 0.37 ± 0.02 dyn/cm^2^, respectively (*p* < 0.05, Figure 1F). The shear stress was higher for 2000 cycles of VUS stimulation (0.37 ± 0.02 dyn/cm^2^) than for 10, 100, and 1000 cycles: 0.11 ± 0.05, 0.11 ± 0.04, and 0.29 ± 0.02 dyn/cm^2^, respectively (*p* < 0.05, Figure 1G). Time-resolved analyses of particle movements under FUS stimulation (acoustic pressure = 500 kPa) (Figure 1D,E) showed that shear stress rapidly reached a value of 0.053 ± 0.003 dyn/cm^2^ at 10 s after applying the stimulation, which was then maintained with no significant difference between 20 s (0.058 ± 0.000 dyn/cm^2^) and 30 s (0.056 ± 0.000 dyn/cm^2^) poststimulation (*p* > 0.05). In contrast, the VUS-induced shear stress increased sharply immediately after stimulation and required up to 30 s to reach a plateau at 0.37 ± 0.02 dyn/cm^2^. The shear stresses at 10 and 20 s poststimulation reached 0.13 ± 0.03 and 0.23 ± 0.02 dyn/cm^2^, respectively (Figure 1H). Together, these findings demonstrate that the VUS system produces high-magnitude, transient shear stress and, hence, represents a promising approach for the mechanical stimulation of ciliated cells.

**Figure 1:**
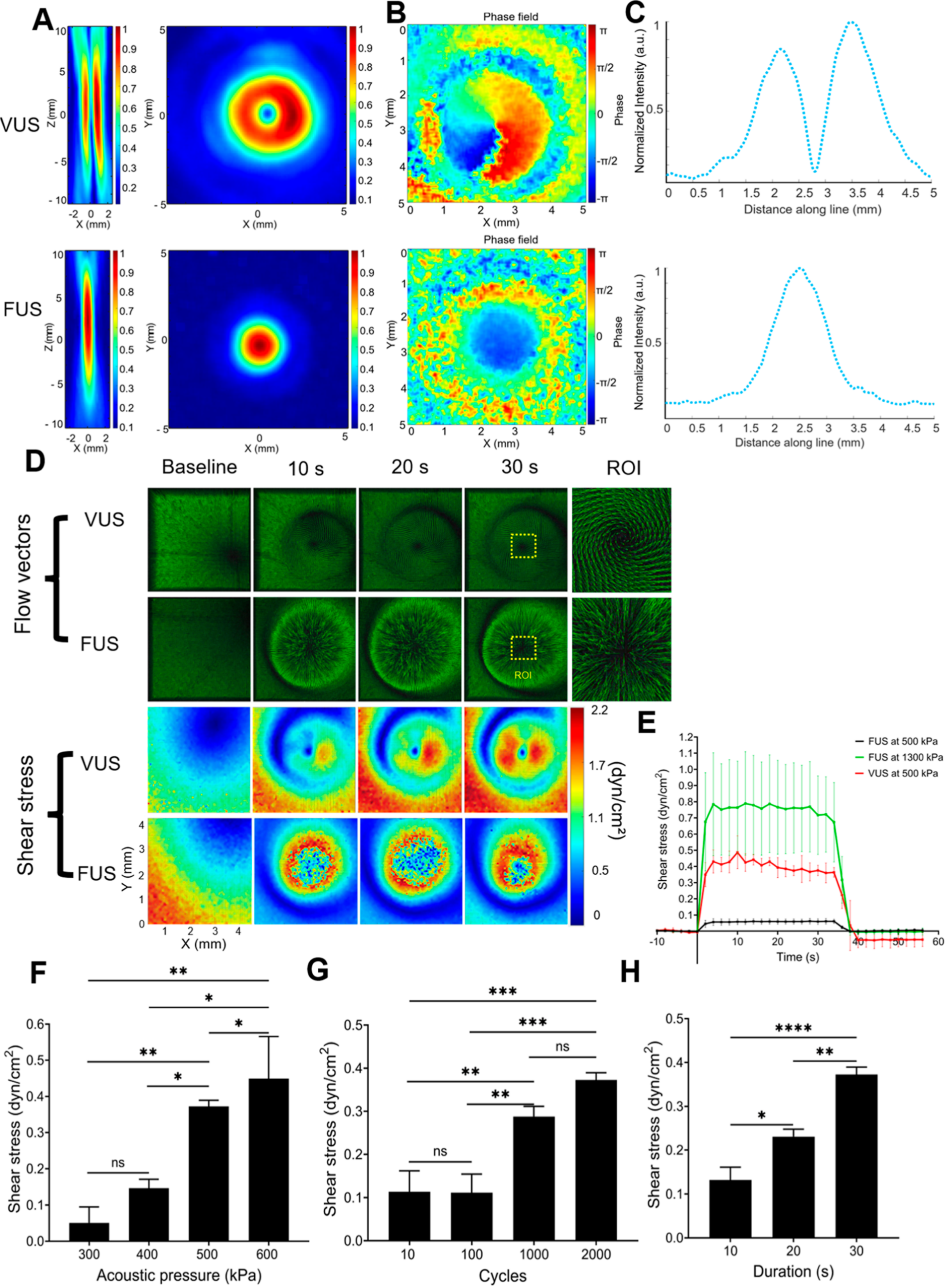
Physical properties of the VUS transducer. (A) FUS and VUS acoustic fields in *xz* planes (left) and *xy* planes (right) at the focal point. (B) Phase fields of the acoustic fields of FUS and VUS in the *xy* plane at *z* = 50 mm (focal depth). (C) Normalized acoustic pressure at the cross-section corresponding to the distribution shown in panel B. (D) Flow vectors at the focal point before and during FUS and VUS stimulation at different time points. (E) Ultrasound-induced shear stress over time with different waveforms (*n* = 3). (F–H) Average shear stress over time during VUS stimulation with different parameters (*n* = 3 per condition). Data are mean ± SEM (ns, not significant; *p < 0.05; **p < 0.01; ***p < 0.001; ****p < 0.0001).

### VUS-Induced Shear Stress Induces Cilia Motility and Cilia-dependent Calcium Influx

We employed mouse inner medullary collecting duct-3 (IMCD3) cells, which express stably SSTR3-GFP (green fluorescent protein fused somatostatin receptor 3) as a model system to evaluate the effects of shear stress on primary cilium motility and to optimize the VUS stimulation parameters (Figure 2A). IMCD3-SSTR3-GFP cells were cultured under serum-starvation medium [0.5% fetal bovine serum (FBS)] to promote ciliogenesis for 24 h. Under these conditions, the cells produced well-defined primary cilia that were marked by SSTR3-GFP. Both the proportion of ciliated cells (65.41 ± 3.88%) and the cilia length (4.58 ± 0.18 μm) were significantly higher than for the control cells maintained in medium containing 10% FBS (34.26 ± 4.48% and 2.70 ± 0.13 μm, respectively) (Figure 2A,B).

**Figure 2:**
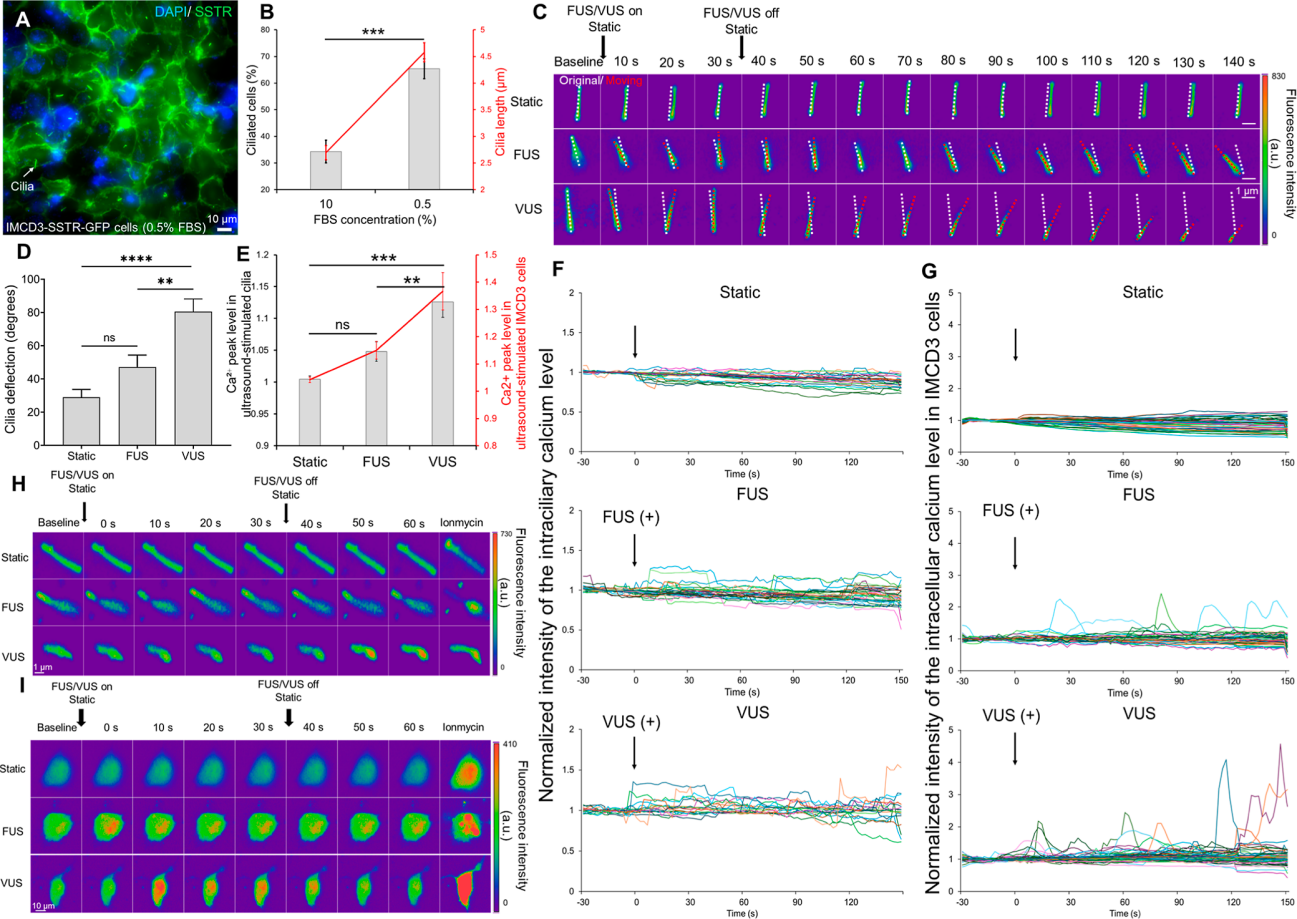
VUS-induced shear stress induces cilia motility that results in calcium influx. (A) Under serum-starvation conditions, IMCD3 cells transfected with SSTR3 exhibit SSTR3-positive primary cilia (green); the nuclei of IMCD3 cells are indicated by DAPI (blue). (B) Both the cilia length and the proportion of ciliated cells were higher after culturing in serum-starvation medium than in the control group (DMEM + 10% FBS) (*n* = 100, 3 independent experiments). (C) Cilia deflections under static, FUS, and VUS stimulation conditions. The cilia are indicated by the rainbow colors, with the white dotted line showing the original position of cilia before stimulation and the red dotted line showing the cilia position after stimulation (static, FUS, and VUS conditions) at different time points. (D) Cilia deflection angles. (E) Ca^2+^ peak level in the cilia (*n* = 25, three independent experiments) and cytosol (*n* = 70 cells, ≥ 3 independent experiments) of ultrasound-stimulated IMCD3 cells. Relative calcium readout over time has been measured every 2 s for 270 s. Data are mean ± SEM (ns, not significant; **p < 0.01; ***p < 0.001; ****p < 0.0001). (F) Normalized intensity of intraciliary calcium influx. (G) Normalized intensity of intracellular calcium influx. (H) Images of intraciliary calcium influx under static, FUS, and VUS stimulation conditions at different time points. (I) Images of cytosolic calcium influx under static, FUS, and VUS stimulation conditions at different time points.

We assessed the effect of VUS-induced shear stress on cilia motility by integrating the VUS system with a live-cell fluorescence microscopy platform (Figure S2A). The SSTR3-GFP signals permitted continuous real-time tracking of ciliary morphology and motility during stimulation. The primary cilia were stimulated by VUS-induced shear stress (acoustic pressure = 500 kPa, 2000 cycles, PRF = 70 Hz; 0.37 ± 0.02 dyn/cm^2^) for 30 s, and the cilia deflection angles were measured as a quantitative index of motility (Figure S3). VUS stimulation induced pronounced cilia deflection of 80.5 ± 7.6° relative to the baseline, corresponding to a tip displacement of 5.17 ± 0.28 μm. In contrast, conventional FUS stimulation under identical acoustic parameters (acoustic pressure = 500 kPa, 2000 cycles, PRF = 70 Hz; 0.050 ± 0.008 dyn/cm^2^) elicited a significantly smaller deflection (47.1 ± 7.2°) and tip displacement (3.12 ± 0.32 μm) (*p* < 0.05) (Figures 2F and S2B). Under the normal physiological (static) condition, SSTR3-GFP-labeled cilia exhibited small spontaneous movements, with an average deflection angle of 29.9 ± 4.8° (tip displacement = 2.22 ± 0.33 μm), which was markedly lower than that induced by VUS (*p* < 0.05), but not significantly different from the FUS-induced motility (*p* > 0.05). Time-resolved live imaging revealed that the cilia deflection increased progressively during stimulation, reaching a maximum at 110 s following VUS onset (80.5 ± 7.6°, Figure 2C). The FUS-induced cilia motility peaked earlier, after approximately 92 s (47.1 ± 7.2°). Upon the cessation of VUS stimulation, the cilia rapidly returned to their baseline position (Video S1). Repeated VUS stimulation produced consistent and reproducible cilia deflection angles (Video S2), confirming the reliability and specificity of the VUS-induced mechanical stimulation.

Mechanically deflecting primary cilia induces the influx of calcium from the extracellular environment through ion channels in the ciliary membrane,^6,41,42^ resulting in increases in both intraciliary and intracellular calcium levels. We therefore used intraciliary and intracellular calcium dynamics as functional indicators of ciliary mechanotransduction following VUS stimulation. The calcium responses within primary cilia were monitored directly using 5HT6 (5-hydroxytriptamine receptor 6)-mCh-G-GECO, which is a genetically encoded ciliary calcium indicator (Figure S2C).^43^ VUS significantly increased the intraciliary calcium level (1.13 ± 0.02 arbitrary units [a.u.]) relative to both FUS stimulation (1.05 ± 0.02 au) and the static condition (1.00 ± 0.00 au, *p* < 0.05) (Figure 2E,F,H).

Because intracellular calcium signaling has been identified as a key downstream response to shear stress,^44,45^ we next quantified cytosolic calcium dynamics using genetically encoded indicator R-GECO. FUS-induced shear stress at 0.050 ± 0.008 dyn/cm^2^ elicited a modest but rapid increase in intracellular calcium to a peak of 1.15 ± 0.03 au, compared with 1.04 ± 0.01 au in the static condition. In contrast, VUS-induced shear stress at 0.37 ± 0.02 dyn/cm^2^ induced a significantly larger intracellular calcium response, peaking at 1.37 ± 0.07 au (*p* < 0.05, Figure 2E,G,I). Together, these results demonstrate that VUS-induced shear stress increases primary cilium motility and also intraciliary and intracellular calcium influx. Compared with conventional FUS, VUS elicits greater cilia deflection and hence more-effective physiological activation of primary cilia.

### Primary Cilia Function as Mechanosensory Organelles for VUS-Induced Shear Stress in Epithelial Cells and Primary Neurons

To verify whether primary cilia serve as the mechanosensory organelles responsible for VUS-induced intracellular calcium elevation, we compared calcium responses under the following four conditions: cilia + static, nonciliated + VUS, cilia + VUS, and cilia + Roscovitine + VUS (Figure 3A). Roscovitine is a pharmacological inhibitor that reduces ciliogenesis.^46^ To validate its efficacy, IMCD3 cells were cultured under serum-starvation medium (0.5% FBS) in the addition of 5 μM Roscovitine for 24 h before applying VUS mechanical stimulation. Quantitative analyses confirmed that Roscovitine markedly reduced ciliogenesis, with the proportion of ciliated cells in the Roscovitine-treated group decreasing to 28.3 ± 2.4%, compared with 65.41 ± 3.70% in cells cultured in serum-starvation conditions and 34.26 ± 4.30% in control cells maintained in 10% FBS (*p* < 0.05, Figure S4A). In addition, the cilia were markedly shorter in the Roscovitine-treated group (2.59 ± 0.08 μm) than in the serum-starvation group (4.58 ± 0.18 μm), but they were of comparable length to those observed under the 10% FBS condition (2.69 ± 0.13 μm, Figure S4B). We next examined intracellular calcium responses in IMCD3 cells under these conditions (Figure 3B). Under the static condition, the intracellular calcium levels remained near the baseline (1.040 ± 0.003 au) and did not differ significantly from those observed in nonciliated cells stimulated by VUS-induced shear stress (1.03 ± 0.02 au). In contrast, cells cultured under serum-starvation conditions that promote ciliogenesis exhibited a marked increase in intracellular calcium upon VUS stimulation, with peak calcium levels reaching 1.32 ± 0.61 au (*p* < 0.001). Notably, inhibition of ciliogenesis by Roscovitine abolished the VUS-induced calcium response, reducing the intracellular calcium level to near the baseline value (1.030 ± 0.002 au). These results indicate that a high density of primary cilia is a critical determinant of intracellular calcium influx in response to VUS-induced shear stress.

**Figure 3:**
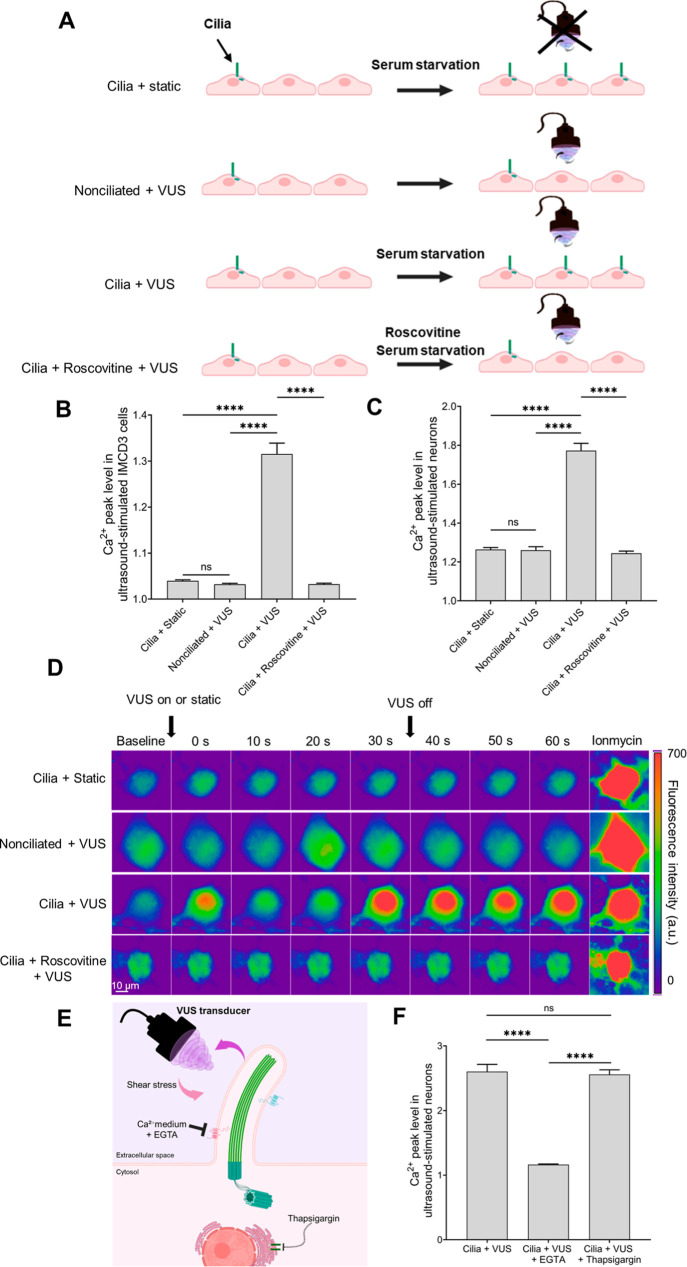
Primary cilia are mechanosensors that respond to VUS-induced shear stress. (A) Diagram of intracellular calcium in IMCD3 cells and primary neurons treated under four conditions: cilia + static, nonciliated + VUS, cilia + VUS, and cilia + Roscovitine + VUS (Roscovitine at 5 μM). The G0/G1 phase of cells was arrested by serum-starvation medium, which induced the primary cilia assembly. Roscovitine inhibits cyclin-dependent kinases, interfering with centriole assembly and thereby suppressing cilia formation. (B, C) Quantification of relative calcium levels in IMCD3 cells (B) and primary neurons (C) over time. The calcium probe signal was acquired for 30 s before treatment as the baseline, 30 s during treatment, and 2 min after treatment before adding the control calcium indicator (10 μM ionomycin). The images were acquired every 2 s for 270 s. Data are mean and SEM values (*n* = 250, ≥ 3 independent experiments). (D) Imaging of intracellular calcium influx in neurons under different stimulated conditions. (E) Scheme of intracellular Ca^2+^ in reply to VUS-induced shear stress with and without the presence of thapsigargin (100 nM) or EGTA (10 μM). The figure was created using Biorender software. (F) Quantification of intracellular Ca^2+^ signaling. Data are mean and SEM values (*n* = 250, ≥ 3 independent experiments) (ns, not significant; ****p < 0.0001).

To assess whether this mechanosensory role of cilia is conserved in neurons, we performed analogous experiments using primary neurons (Figure 3C,D). Consistent with the observations in IMCD3 cells, the intracellular calcium response was strongest in ciliated neurons exposed to VUS (1.77 ± 0.04 au), with significantly lower responses detected in the cilia + static (1.27 ± 0.01 au), nonciliated + VUS (1.26 ± 0.02 au), and cilia + Roscovitine + VUS (1.25 ± 0.01 au) conditions (*p* < 0.001). These findings demonstrated that primary cilia similarly function as mechanosensors for VUS-induced shear stress in neurons.

To determine the origination of the calcium signals underlying the neuronal response, we next disrupted intracellular calcium stores by using thapsigargin, a chemical suppresses the sarco-/endoplasmic reticulum Ca^2+^ ATPase (SERCA) (Figure 3E,F). Depleting the intracellular calcium stores did not significantly reduce the peak intracellular calcium response to VUS-induced shear stress in ciliated neurons (2.60 ± 0.11 au in controls and 2.56 ± 0.07 au with thapsigargin treatment). In contrast, stimulating neurons with VUS in calcium-free medium supplemented with 10 μM EGTA markedly reduced the intracellular calcium response, with the peak level reduced to 1.16 ± 0.00 au (Figure 3F). These results indicate that VUS-induced calcium signaling primarily arises from the intracellular influx of calcium from the extracellular medium rather than release from intracellular stores.

Together, these results demonstrate that VUS-induced shear stress activates intracellular calcium signaling in both epithelial cells and primary neurons via the ciliary mechanotransduction mechanism. Primary cilia functions as the key mechanosensory organelles that couple localized shear stress to mediate a significant rise of calcium influx, which flows from the extracellular medium to the intracellular area.

### Magnitude of VUS Stimulation Determines the Magnitude of Cilia Motility

To determine the optimal VUS parameters for inducing primary cilium motility, ciliated IMCD3-SSTR3-GFP cells were stimulated across a range of acoustic conditions (acoustic pressure = 300–600 kPa, 10–2000 cycles, PRF = 70 Hz, stimulation duration = 10–30 s). The cilia deflection angle and intracellular calcium responses were quantified as complementary indicators of ciliary mechanotransduction. Cilia deflection increased with the magnitude of VUS stimulation and peaked in cells exposed to VUS at an acoustic pressure of 500 kPa (3.5 MHz transducer, 2000 cycles, PRF = 70 Hz; shear stress = 0.37 ± 0.02 dyn/cm^2^), which induced a maximum deflection angle of 91.70 ± 13.20°. Although the differences among pressure groups did not reach statistical significance, a clear trend was observed, with acoustic pressures of 300, 400, and 600 kPa (corresponding to stresses of 0.05 ± 0.04, 0.15 ± 0.02, and 0.45 ± 0.12 dyn/cm^2^, respectively) inducing deflections of 65.21 ± 9.10, 70.30 ± 15.40, and 83.20 ± 10.70°, respectively (*p* > 0.05; Figure 4A). Similarly, increasing the number of cycles increased the cilia deflection, with the deflection being larger at 2000 cycles (0.37 ± 0.02 dyn/cm^2^, 91.70 ± 13.20°) than those at 10, 100, and 1000 cycles (0.11 ± 0.05, 0.11 ± 0.04, and 0.29 ± 0.02 dyn/cm^2^, respectively): 34.30 ± 4.92, 70.10 ± 12.40, and 67.10 ± 10.50°, respectively (Figure 4B). The cilia deflection was also dependent on the stimulation duration: although cilia responded after 10 and 20 s of VUS exposure (56.30 ± 7.60 and 62.70 ± 9.70°, respectively), the deflection magnitude was substantially larger after 30 s (91.70 ± 13.20°) (Figure 4C). Together, these results indicate that sustained VUS-induced shear stress is required to reliably elicit cilia motility.

**Figure 4:**
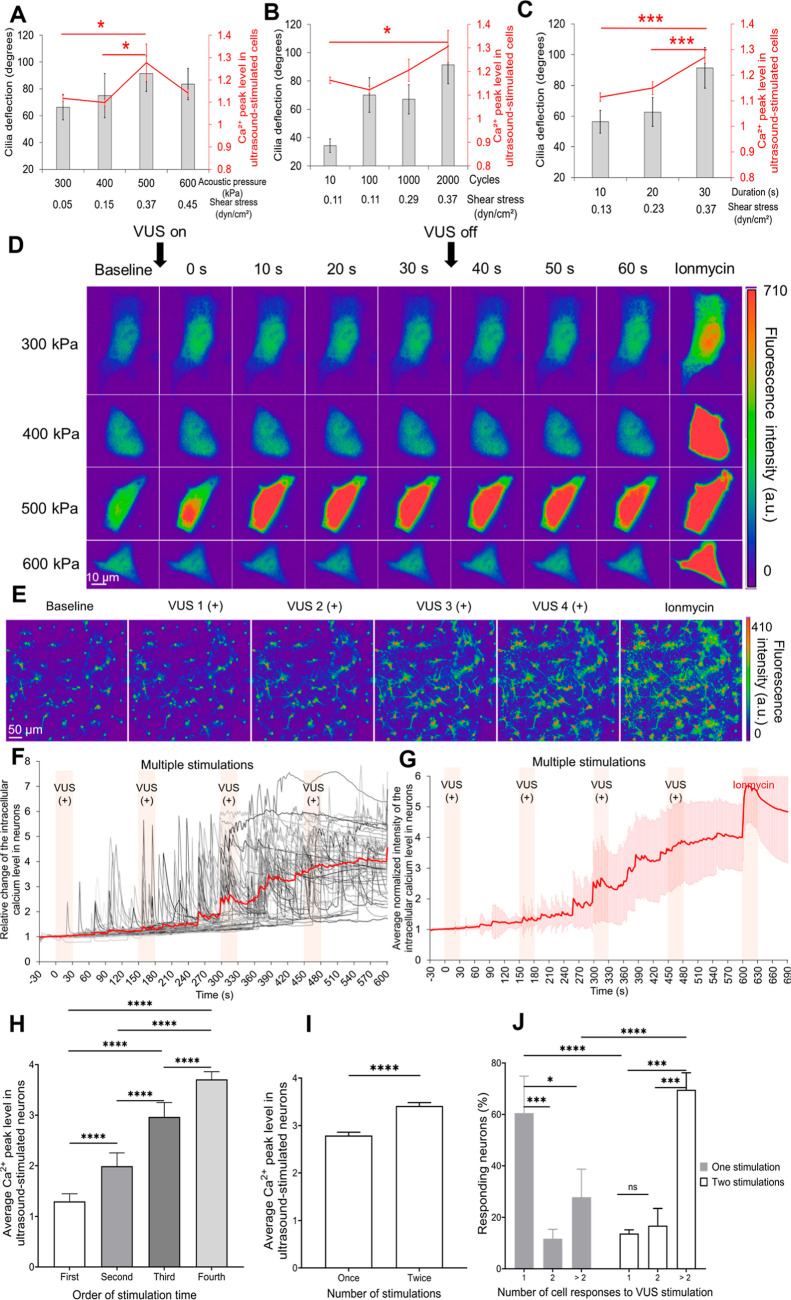
VUS magnitude modulates cilia motility and Ca^2+^ responses. (A–C) Correlations between ciliary deflection angle and Ca^2+^ signals under varying VUS parameters (mean and SEM values, *n* = 70 cells, ≥ 3 independent experiments; *p < 0.05): (A) 3.5 MHz transducer, acoustic pressure = 300–600 kPa, 2000 cycles, PRF = 70 Hz, stimulation duration = 30 s; (B) 3.5 MHz transducer, acoustic pressure = 500 kPa, 10–2000 cycles, PRF = 70 Hz, stimulation duration = 30 s; and (C) 3.5 MHz transducer, acoustic pressure = 500 kPa, 2000 cycles, PRF = 70 Hz, stimulation duration = 10–30 s. (D) Time-lapse microscopy of IMCD3 cells showing VUS-induced Ca^2+^ influx at an acoustic pressure of 300–600 kPa (3.5 MHz transducer, 2000 cycles, PRF = 70 Hz, stimulation duration = 30 s). Arrows indicate VUS onset (30 s) and cessation (60 s). Ionomycin (10 μM) served as a positive control. (E) Ciliated neurons were imaged 30 s before stimulation (baseline) and after four consecutive VUS-induced shear-stress pulses (0.37 ± 0.02 dyn/cm^2^; 30 s each; 120 s intervals). Ionomycin was used as a positive control. (F) Normalized neuron intracellular Ca^2+^ dynamics following VUS (*n* = 70 cells; gray, individual cells; red, mean). (G) Mean normalized neuron Ca^2+^ intensity (data are mean and SD values, *n* = 250 cells, ≥ 3 experiments). (H) Mean Ca^2+^ peak level after VUS (30 s stimulation, 120 s poststimulation; mean ± SEM, *n* = 250 cells, ≥ 3 experiments; ***p < 0.0001). (I,J) Ca^2+^ peak level after one or two stimulations and percentage of responsive neurons (mean and SEM values, *n* = 250 cells, ≥ 3 experiments; ns, not significant; *p < 0.05; ***p < 0.001; ****p < 0.0001).

Intracellular calcium measurements mirrored the trends described above. VUS stimulation at an acoustic pressure of 500 kPa (0.37 ± 0.02 dyn/cm^2^) induced the strongest calcium response (1.28 ± 0.08 au), which was significantly larger than the responses observed at acoustic pressures of 300, 400, and 600 kPa (0.05 ± 0.04, 0.15 ± 0.02, and 0.45 ± 0.12 dyn/cm^2^, respectively): 0.15 ± 0.02, 1.10 ± 0.01, and, 1.14 ± 0.02 au, respectively (*p* < 0.05, Figure 4A,D). Likewise, stimulation with 2000 cycles produced the largest calcium increase (1.31 ± 0.06 au) (*p* < 0.05, Figure 4B). A minimum stimulation duration of 30 s was required to induce a significant intracellular calcium increase (1.27 ± 0.02 au) (*p* < 0.05, Figure 4C). These findings suggest that a threshold mechanical stimulation needs to be exceeded to achieve effective VUS-induced activation of primary cilia.

Importantly, the cell viability assays revealed no significant cytotoxic effects across all of the VUS parameter combinations tested (*p* > 0.05, Figure S5A–C), nor any evidence of ciliary disassembly (*p* > 0.05, Figure S5D–F). Based on these analyses, VUS parameters of an acoustic pressure of 500 kPa, 2000 cycles, PRF of 70 Hz, and a stimulation duration of 30 s were selected as optimal conditions for the subsequent experiments.

### Repeated VUS Stimulation Increases Neuronal Calcium Responses

We next examined how ciliated neurons respond to repeated VUS-induced shear stress using the optimal stimulation parameters (3.5 MHz transducer, acoustic pressure = 500 kPa, 2000 cycles, PRF = 70 Hz, stimulation duration = 30 s; 0.37 ± 0.02 dyn/cm^2^) (Figure 4E–G). Neurons exhibited progressively increased intracellular calcium responses upon repeated VUS stimulation. Following a second stimulation, the cytosolic calcium level increased to 2.00 ± 0.03 au, representing an approximately 1.5-fold increase relative to the first stimulation (1.30 ± 0.02 au, Figure 4H), without compromising neuron viability (105.0 ± 1.6%, 98.7 ± 1.9%, and 98.8 ± 2.6% for no stimulation, one stimulation, and two stimulations, respectively) (Figure S6). However, more stimulations resulted in excessive calcium accumulation (3.00 ± 0.03 and 3.70 ± 0.02 au for three and four stimulations, respectively), accompanied by marked reductions in neuron viability (81.8 ± 2.6% and 71.4 ± 2.6%, respectively). To distinguish whether calcium accumulation resulted from sustained signaling or from residual responses, neurons were stimulated once or twice and monitored for 10 min poststimulation. A single stimulation induced a transient calcium increase (maximum of 2.63 ± 0.09 au) that returned to near the baseline, whereas two stimulations produced a significantly larger and more sustained calcium response (3.56 ± 0.10 au, Figure 4I). Repeated VUS stimulation increased both the magnitude and occurrence of calcium responses. After a single stimulation, 70% of neurons exhibited a single calcium transient, whereas only 21% displayed three or more responses. In contrast, following two stimulations, 72% of neurons exhibited three or more calcium transients (*p* < 0.05, Figure 4J). Together, these results demonstrate that repeated VUS-induced shear stress increases neuronal calcium signaling in a cumulative and dose-dependent manner, while still maintaining neuron viability within a defined stimulation window.

### TRPV4 and TRPP2Mediate Cilia-Dependent Calcium Influx in Response to VUS-Induced Shear Stress

To further characterize the molecular mechanisms underlying ciliary mechanotransduction in response to VUS-induced shear stress, we used pharmacological inhibitors to investigate the contribution of specific ciliary membrane ion channels to the observed responses (Figure 5A). The intracellular calcium influx was used as the primary functional indicator of channel activation. We focused on candidate mechanosensitive channels that have previously been implicated in ciliary signaling, including transient receptor potential (TRP) vanilloid 4 (TRPV4), TRPC1 (TRP canonical 1), and TRPP2/3.^43,47–49^

**Figure 5:**
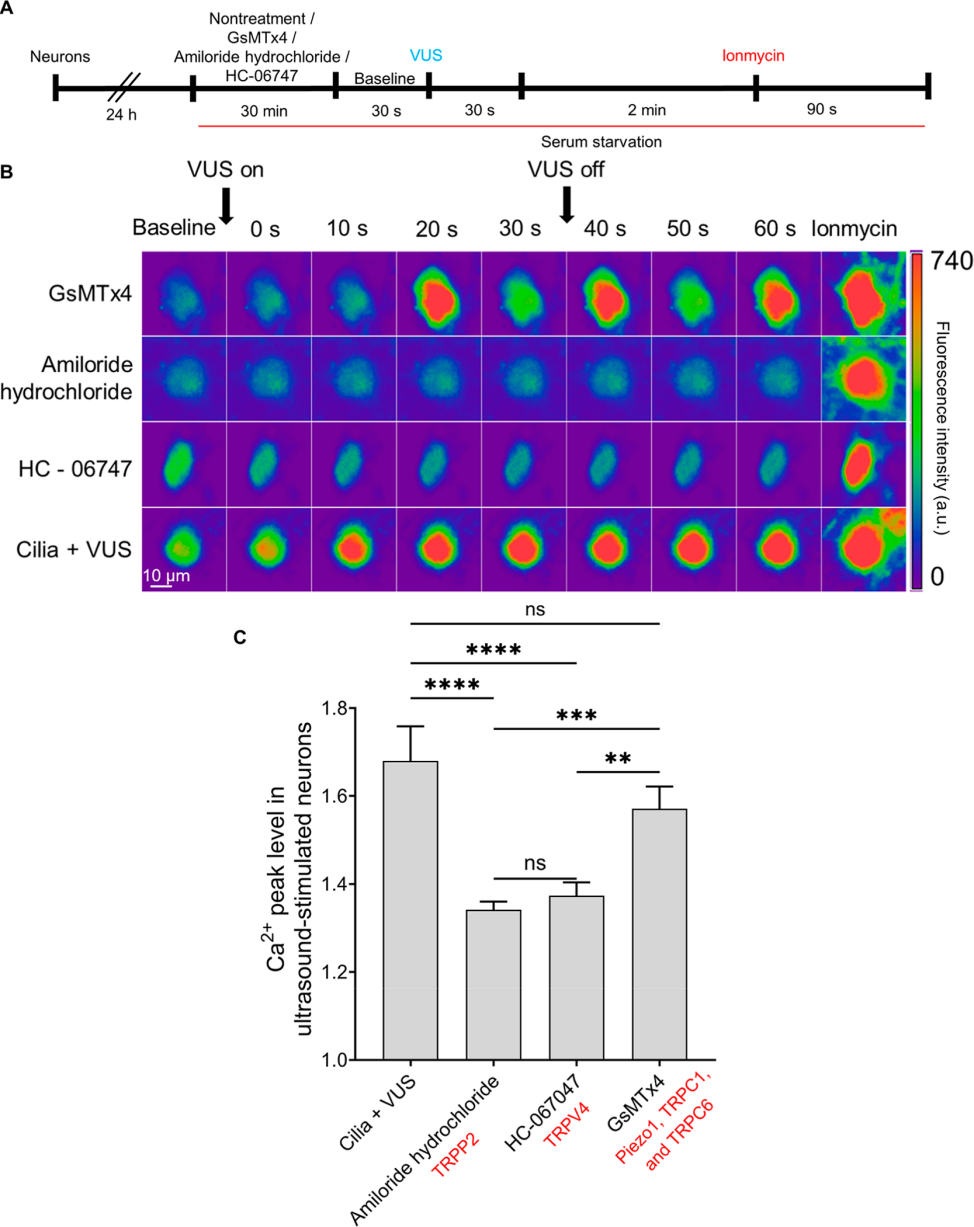
Responses of primary cilia on neurons to VUS-induced shear stress as indicated by calcium influx mediated by ciliary ion channels. (A) Experimental schedule. The role of cilia ion channels in responses to VUS-induced shear stress was assessed by comparing the calcium influx in ciliated neurons treated with ion-channel blockers (GsMTx4, amiloride hydrochloride, and HC-067047) with the untreated group. Neurons were promoted ciliary elongation by being maintained in serum-starvation medium (0.5% B-27) for 24 h before either not treated or exposed to GsMTx4 (2.5 μM), amiloride hydrochloride (1 μM), or HC-067047 (1 μM) for 30 min before experimentation. Chemically treated neurons were imaged for a 30 s baseline period before stimulation with VUS-induced shear stress. Time-lapse imaging was performed for 150 s during and after VUS stimulation. Ionomycin was used as a calcium positive control. (B) Time-lapse microscopy images. (C) Calcium peak level in neurons stimulated by VUS-induced shear stress. Data are mean and SEM values (*n* = 250 cells, ≥ 3 independent experiments) (****p < 0.001). Red words indicate ion channels blocked by the corresponding chemicals. Amiloride hydrochloride is an inhibitor of TRPP2. GsMTx4 is a broad inhibitor of mechanosensitive cation channels (Piezo1, TRPC1, and TRPC6). HC-067047 is a selective TRPV4 antagonist.

We first applied GsMTx4, which is a broad inhibitor of mechanosensitive cation channels, including Piezo1 and certain TRP channels.^50^ Treatment with GsMTx4 did not significantly reduce the VUS-induced intracellular calcium response in ciliated neurons (1.49 ± 0.49 au with GsMTx4 versus 1.38 ± 0.10 au in untreated controls; Figure 5B,C), suggesting that these channels are not major contributors to VUS-induced mechanotransduction.

We next examined the role of polycystin channels using amiloride hydrochloride, which is a pharmacological inhibitor that reduces TRPP2-dependent calcium influx.^51^ In contrast to GsMTx4, amiloride treatment significantly reduced the VUS-induced calcium response in ciliated neurons, decreasing the peak intracellular calcium level from 1.49 ± 0.49 to 1.20 ± 0.03 au (Figure 5B,C).

To apply the selective TRPV4 antagonist HC-067047 to assess the involvement of the TRPV4 mechanosensitive calcium-permeable channel that is abundant in primary cilia.^52^ Inhibition of TRPV4 markedly reduced the intracellular calcium level from 1.49 ± 0.49 au to near the baseline value of 1.07 ± 0.02 au (Figure 5B,C).

Collectively, these results indicate that VUS-induced shear stress is transduced at the neuronal cilium primarily via TRPV4- and TRPP2-dependent calcium influx, with Piezo1-sensitive and other GsMTx4-sensitive channels playing minimal roles. These findings identify TRPV4 and TRPP2 as key mechanosensitive ion channels mediating ciliary calcium signaling in association with VUS-induced shear stress.

## Discussion

Primary cilia are ultrastructural organelles found in a wide range of mammalian cells, including epithelial cells, osteocytes, chondrocytes, neurons, and endothelial cells.^4,53–57^ Cilia functions across diverse organ systems as mechanosensitive structures that detect shear stress generated by fluid flow in the body. Disruption of the ciliary structure or function is associated with multiple pathological conditions, including hydrocephalus and scoliosis.^58–60^ The present study has demonstrated that VUS regulates ciliary mechanotransduction in both neurons and epithelial cells by generating localized shear stress. Our findings show that VUS-induced shear stress increases cilia motility and triggers downstream cellular responses, most notably calcium signaling. We have further elucidated the mechanisms underlying the sensitivity of neuronal cilia to shear stress induced by VUS stimulation, which opens the potential for VUS-induced shear stress in neuromodulation.

This study demonstrated that VUS and FUS stimulation can induce shear stress, with the VUS-induced shear stress (0.37 ± 0.02 dyn/cm^2^) being 5-fold higher than FUS-induced shear stress. The VUS-induced shear stress also exceeded both the flow-induced forces in vivo to activate cilia motility and the force produced by optical tweezers used to stimulate cilia motility (0.1 and 0.6 pN, respectively),^61,62^ while remaining well below pathological shear stresses (16.6 dyn/cm^2^).^63^ Therefore, VUS-induced shear stress may be a powerful tool for displacing primary cilia in neurons, with this displacement depending on the magnitude of the generated shear stress, thereby increasing their functional activity. VUS stimulation induced a larger cilia deflection angle (80°) that was maintained for a longer time (110 s) compared with using FUS (47° and 92 s, respectively). However, it was particularly interesting that there was a delay before the cilia responded to VUS stimulation compared with FUS stimulation. This can be explained by the results obtained for VUS-induced shear stress when comparing different stimulation durations (10–30 s). FUS- induced shear stress showed no significant difference between 10, 20, and 30 s (*p* > 0.05), which is suitable for the theory that under ideal steady-flow conditions, shear stress depends on viscosity and shear rate and is time-independent.^64^ However, VUS generates tornado-shaped translational Rayleigh acoustic streaming (unsteady flow) in the vicinity of the oscillating boundary.^36^ It is a time-dependent movement, with a small hysteretic displacement when the fluid changes from the quiescent condition at the onset of acoustic streaming to reach its maximum amplitude value before remaining constant.^65^ Therefore, VUS induces an acoustic streaming force, which is a time-dependent movement, and so VUS stimulation requires time to generate sufficient shear stress to move the cilia. Inertial forces result in the cilia motility continuing even after the VUS stimulation has stopped. Meanwhile, FUS generates only translational movement, and the shear stress is significantly lower than for VUS. This results in the cilia deflections being smaller and being maintained for a shorter time. Further results comparing cilia motility under VUS stimulation for different durations (10–30 s) also confirmed this finding. Cilia motility after stimulation by VUS-induced shear stress produced a rapid increase in calcium concentrations in both the cilia and the cytoplasm. Our results are similar to those of Rydholm et al.,^66^ who stimulated cilia with shear stress from a Plexiglas cylinder. They found that the stress increased only gradually, taking more than 20 s to reach its maximum value after the force was applied, and that there was a delay of approximately 20 s before the epithelial cells responded. In addition, a single pulse with a duration of 30 s was required to stimulate cilia deflection, with shorter pulses (5 s) not eliciting responses from ciliated cells.

We also observed that cells under low-serum conditions exhibited larger Ca^2+^ responses than nonstarved cells for stimulation by VUS-induced shear stress. The disruption of the cilia assembly by Roscovitine reduces the cellular response to VUS-induced shear stress. This might be due to serum-starvation-induced synchronization increasing the proportion of cells expressing primary cilia, indicating a determinant role for cilia in modulating calcium signaling. It was considered unlikely that the endoplasmic reticulum was the principal store of calcium release under VUS stimulation. However, experiments showed that thapsigargin-induced depletion of intracellular calcium stores did not significantly reduce the intracellular calcium response. Furthermore, EGTA-mediated depletion of extracellular calcium reduced these signals, indicating that the mechanosensitive response to shear stress depends on calcium influx from the extracellular medium. These results indicated that the cytosolic calcium increase induced by VUS stimulation is primarily caused by cilia deflection, which allows calcium ions (Ca^2+^) from the extracellular environment to enter the cell.

The use of different VUS parameters when stimulating cilia resulted in varying deflections that led to distinct downstream cell responses. The efficacy was greatest when applying an acoustic pressure of 500 kPa, 2000 cycles, and a stimulation duration of 30 s (0.37 ± 0.02 dyn/cm^2^). However, the VUS parameters did not affect cell viability, nor did the cilia assembly ratio, calculated as the percentage of cilia after VUS stimulation relative to that before stimulation. This demonstrates that VUS can be used to safely control cilia motility. Repeated stimulation by VUS-induced shear stress resulted in the neuronal response accumulating with each stimulation. However, after three or four stimulations, it was evident that calcium had leaked from the intracellular area into the extracellular environment via the cell membrane, and the cell viability decreased significantly. Meanwhile, after two VUS stimulations, the neuronal response to shear stress was significantly larger than for a single stimulation, without affecting cell viability. This result is also in line with the results of Maneshi et al.,^67^ who repeatedly stimulated astrocytes with pulses at an acoustic pressure of 11.5 dyn/cm^2^. They found that the mechanical effect was cumulative during continuous stimulation, with the cell response being 4-fold higher than for a single pulse of the same magnitude and total stimulation duration, suggesting that internal stress might require time to relax.

Multiple calcium channels are involved in primary cilium–mediated mechanotransduction and are expressed in neurons. For example, the membrane of primary cilia consists of diverse calcium channels, including members of the TRP family.^68^ Cilia deflection induced by fluid flow promotes the intracellular influx of calcium through these channels that triggers downstream signaling pathways that have been shown to depend on ciliary TRPP2. TRPP2 is an ion channel found in primary cilia of kidney cells, neurons, and vascular endothelial cells.^6,49,51,68^ However, its function has been demonstrated to be activated by the TRPV4 ion channel, since it is not a mechanoreceptor. TRPV4 connects TRPP2 to form a mechanosensitive molecular sensor^69^ that has been demonstrated to promote significant potassium release and Ca^2+^ influx in epithelial cells,^70^ thereby inducing cellular responses by triggering downstream signaling pathways. This means that TRPP2 and TRPV4 engage in physical and functional responses to fluid-flow-induced shear stress. These results suggest that primary cilia in murine neurons sense VUS-induced shear stress and transduce this mechanical stimulus into a calcium-dependent signaling pathway, potentially through the activation of specific ion channels (e.g., TRPV4 or TRPP2) and hence the regulation of neuronal physiological processes. These results demonstrate that TRPP2 and TRPV4 are vital components of the ciliary fluid-flow-induced shear stress sensor in primary neurons.

This study has also shown that primary cilia contribute to the mechanical sensitivity of neurons via mechanotransduction. In addition, RNA sequencing results (unpublished) indicate that interactions between TRPP2 and TRPV4 in response to VUS-induced shear stress increase the expression of proteins involved in the responses to mechanical stimuli, as well as proteins related to cell proliferation and differentiation, and stimulate angiogenesis, thereby promoting downstream signaling pathways and opening up further potential applications of the VUS stimulation method.

## Conclusions

Our study establishes that 30 s of VUS stimulation at 3.5 MHz generates a shear stress of 0.37 ± 0.02 dyn/cm^2^, which induces robust primary cilium deflection and enhances both ciliary and intracellular Ca^2+^ signals. Optimization of acoustic pressure (300–600 kPa), pulse duration (10–2000 cycles), and stimulation time (10–30 s) identified 3.5 MHz, 500 kPa, 2000 cycles, and a 70 Hz pulse repetition frequency as the optimal stimulation parameters. Under these conditions, VUS enhanced ciliary motility and Ca^2+^ signaling without inducing ciliary disassembly or compromising cell viability. Notably, two stimulation sections increased neuronal responses while preserving cell viability, whereas three or more cycles caused marked cytotoxicity. Mechanistically, primary cilia served as the principal mechanosensory organelles, with the ciliary ion channels TRPV4 and TRPP2 mediating mechanotransduction from VUS-induced shear stress by facilitating extracellular Ca^2+^ influx, thereby initiating intracellular calcium signaling and modulating neuronal responses. Together, these in vitro findings provide a mechanistic foundation for VUS-mediated ciliary mechanotransduction and establish VUS as a noninvasive, deep-penetrating, high spatial-resolution, and high-shear-stress platform for targeted neuromodulation in the future.

## Experimental Methods

### Cell Culture

The IMCD3 cells were cultured in Dulbecco’s modified Eagle’s medium (DMEM; 10–013-CV, Corning), added with 10% FBS (35–010-CV, Corning) and 1% penicillin–streptomycin (30–002-CI, Corning) at 37 °C, 5% CO_2_.

### Primary Neuron Culture

Primary cortical neurons were prepared from embryonic day 18 Sprague–Dawley rat embryos obtained from the National Center for Biomodels (NCB, NIAR, Taiwan). Cortices were carefully dissected and immediately washed with calcium- and magnesium-free Hank’s balanced salt solution (HBSS; 14185–052, Gibco) supplemented with 1 M HEPES buffer (15630–080, Gibco) to reduce potential contamination. Tissue digestion was performed at 37 °C for 15–20 min in a papain-based enzymatic solution consisting of 0.6 mg/mL papain (76220, Sigma-Aldrich, USA), 0.6 mg/mL DNase I (DN25, Sigma-Aldrich), 0.2 mg/mL l-cysteine (173600250, Thermo Fisher Scientific, Waltham, MA, USA), 1.5 mM CaCl_2_ (A2306, Biomatik), and 0.5 mM EDTA (AM9260G, Invitrogen) prepared in HBSS. During incubation, samples were gently agitated to ensure uniform exposure to the digestion buffer. Enzymatic activity was terminated by adding Neurobasal medium (A35829–01, Gibco) supplemented with 10% horse serum (16050, Gibco). Following enzymatic treatment, tissues were mechanically dissociated by gentle trituration (20–30 strokes) using a 10 mL plastic pipet (13–678–11E, Thermo Fisher Scientific). The resulting suspension was passed through a 40 μm cell strainer to remove residual debris and subsequently centrifuged at 1000*g* for 5 min at room temperature. Cell pellets were resuspended in Neurobasal medium containing 1× GlutaMAX (35050–061, Gibco), 12.5 μM l-glutamic acid (G1251, Sigma-Aldrich), 2% B-27 supplement (A35828–01, Gibco), and 1% penicillin–streptomycin. Cells were seeded onto poly-l-lysine–coated six-well plates at a density of 8 × 10^5^ cells per well. Cultures were maintained at 37 °C in a humidified incubator with 5% CO_2_. One-third of the culture medium was replaced every 3–4 days with fresh medium lacking l-glutamic acid.

### DNA Constructs

The plasmid pEGFP-C1 (BD Biosciences Clontech) served as the backbone vector for generating R-GECO, a red fluorescent genetically encoded calcium indicator. For ciliary targeting experiments, the cilia-enriched membrane protein 5HT6 was fused at its cytoplasmic C-terminus with mCherry–G-GECO; this construct was kindly provided by Dr. Takanari Inoue. To establish stable IMCD3 cells expressing SSTR3–GFP, the pEGFPN3-SSTR3 plasmid (Addgene #35623) was subcloned into a puromycin-resistant lentiviral expression vector. Cloning was performed using Q5 High-Fidelity DNA polymerase together with the HiFi DNA Assembly system (New England Biolabs) according to the manufacturer’s instructions.

### Lentivirus Production and Infection

Lenti-X 293T cells (TaKaRa) were maintained in DMEM (Corning) supplemented with 10% (v/v) fetal bovine serum (FBS; Corning), 5 U/mL penicillin, and 50 μg/mL streptomycin (Corning) at 37 °C in a humidified 5% CO_2_ incubator. For virus production, 4 × 10^6^ cells were plated onto 10 cm culture dishes (Thermo Fisher Scientific) 24 h before transfection and allowed to attach overnight. For lentiviral packaging, 7 μg of plasmid DNA was diluted in sterile water to a total volume of 600 μL and combined with the Lenti-X Packaging Single Shots reagent (TaKaRa). The mixture was vortexed to dissolve the lyophilized pellet and incubated at room temperature for 10 min before being added to the cells. Following 4 h of incubation at 37 °C, 6 mL of fresh complete medium was added to each dish. Viral supernatants were harvested 48 h later and clarified by filtration through a 0.45 μm poly­(ether sulfone) membrane. For transduction, IMCD3 cells were seeded at a density of 5 × 10^4^ cells per well in six-well plates (Thermo Fisher Scientific) and cultured overnight. Filtered lentiviral supernatant was applied to the cells, and stable integrants were selected 24 h postinfection using 1 μg/mL puromycin (A11138–03, Gibco). Cells were maintained under continuous puromycin selection thereafter.

### Fabricating IMCD3-SSTR3-GFP Cells

The SSTR3-GFP fragment was amplified by PCR from the pEGFP-SSTR3 construct and then subcloned into the pLAS3w.Pneo vector to generate pLAS3w.Pneo-SSTR3-GFP. The pLAS3w.Pneo vectors were from the RNAi Core Facility (Academia Sinica, Taiwan). Lentiviruses were produced in 293FT cells (R70007, Thermo Fisher Scientific). Briefly, 4 × 10^6^ 293FT cells were seeded on a 100 mm culture dish 1 day before transfection. Cells were cotransfected with 6 μg of VSV-G envelope plasmid, 12 μg of pCMV-dR8.91 packaging plasmid, and 12 μg of pLS3w.Pneo-SSTR3-GFP expression plasmid using T-Pro NTRII transfection reagent (JT97-N002M, T-Pro Biotechnology) in accord with the manufacturer’s instructions. At 48–60 h posttransfection, cell debris in culture supernatants containing lentiviral particles was removed by centrifuging (4000 rpm, 10 min) and filtering through a 0.45 μm filter (16555 K, Sartorius).

For lentiviral infection, 5 × 10^6^ IMCD3 cells were cultured on a 100 mm culture dish and incubated with 2 mL of lentivirus supernatant for 24 h, then replaced with fresh medium. At 48 h after infection, transduced cells were selected using G418 (500 μg/mL).

### VUS Setup and Parameter Selection

A FUS transducer (3.5 MHz, 30 mm in diameter; V380, PANAMETRICS, Olympus, USA) or a VUS transducer was used. The VUS transducer was implemented by combining a planar ultrasound transducer (3.5 MHz, 30 mm in diameter) (V380, PANAMETRICS, Olympus, USA) with a handmade focused vortex lens (the fabrication process is described below) (Figure S1A). The acoustic intensity at the transducer focus was monitored by a calibrated needle-type hydrophone (HNC-0400, ONDA, Sunnyvale, USA) in a degassed water tank, which was controlled by three-axis translation scanning during stimulation by FUS or VUS. The focus had a measured diameter and length of 4 and 22 mm, respectively, for both the FUS and VUS acoustic fields (Figure 1A,C). Both transducers had a focal length of 50 mm. The FUS and VUS pressures at the focal point were measured using a calibrated hydrophone (HGL0085, ONDA, Sunnyvale, USA) in a degassed water tank. For the XY-plane phase map, the measurement plane was positioned at the focal depth of 50 mm from the transducer surface. The scanning area was 10 × 10 mm^2^ and was divided into 101 × 101 measurement points, resulting in a total of 10,201 RF waveforms. Based on this setting, the spatial step size in the XY plane was approximately 0.1 mm in both the X and *Y* directions. In addition, for the XZ-plane acoustic-field measurement, the scanning area was set to 5 × 20 mm^2^ and was also divided into 101 × 101 measurement points. Therefore, the spatial step sizes in the X and *Z* directions were approximately 0.05 mm and 0.2 mm, respectively. These scanning parameters were selected based on the three-axis motorized stage’s scanning time, data storage capacity, signal acquisition stability, and the computer hardware’s computational load. After acoustic-field scanning, all measured RF signals were processed in MATLAB for subsequent analysis. First, a band-pass filter was applied to the RF signals acquired at each scanning position to remove noise outside the target frequency range. The acoustic amplitude map was reconstructed by extracting the peak amplitude of each RF waveform, then normalizing, thereby presenting the relative acoustic pressure distribution. For phase-map reconstruction, the filtered RF signals were converted into analytic signals using the Hilbert transform, and the instantaneous phase was then extracted to reconstruct the spatial phase distribution. The continuous phase transition from – π to π radians was reconstructed from experimentally measured raw RF signals after signal processing. The VUS pulses (Frequency: 3.5 MHz, PRF = 70 Hz, acoustic peak negative pressure = 300–600 kPa [input voltage: 92–239 mVpp], 2000 cycles, stimulation duration = 10–30 s) were provided by a function generator (AFG3251, Tektronix, Beaverton, OR, USA) and using a radio frequency power amplifier (model 2100L, Rochester, NY, USA) with a power gain of 50 dB to deliver to the VUS transducer. In this study, VUS parameters were used related to the planar transducer’s design limitations.

The design of the acoustic lens was based on the phase modulation of the ultrasound source by its thickness profile, which was defined by the desired phase map on the source surface as follows

1 $$T\left(r,\theta \right)=\frac{2\pi -\varphi \left(r,\theta \right)}{k_{m}-k_{h}}$$

where the thickness of the acoustic lens pixel at polar coordinates (*r*, θ) is *T*(*r*, θ), k_m_ and *k*_*h*_ were the wavenumbers of the surrounding medium and the materials for the acoustic lens, respectively, and φ­(*r*, θ) is the phase map for the acoustic patterns of interest, which could be calculated for the focal point as.

2 $$\varphi \left(r,\theta \right)=k_{m}\left(f-\sqrt{f^{2}-r^{2}}\right)$$

where *f* is the distance between the focal point and the transducer surface, and for the vortex as

3 $$\varphi \left(r,\theta \right)=k_{m}\left(f-\sqrt{f^{2}-r^{2}}\right)+\pi -\theta$$

The thickness profile was translated into the 3D structure in standard triangle language (STL) format using SolidWorks 2022, and the acoustic lens was then fabricated from VeroClear (attenuation coefficient at 3.5 MHz: 12.9 dB/cm,^38^ measured acoustic impedance: 2.88 × 10^6^ Pa·s/m), a commonly adopted material for ultrasound modulation.^39,40^ The acoustic lens was printed using a 3D printer (J750, Stratasys, USA), which provided a lateral print resolution of 42 μm (X, *Y* direction) and a vertical resolution of 14 μm (*Z* direction), and these feature sizes were substantially smaller than the half wavelength of the ultrasound in water at 3.5 MHz (λ/2 ≈ 212 μm), ensuring high-fidelity reproduction of the desired phase profiles.

### VUS-Induced Shear Stress

IMCD3 cells were used to determine the biological effects of shear stresses induced by FUS and VUS stimulation. The setup included a metal ring with a glass coverslip containing IMCD3 cells, a FUS or VUS system to induce shear stress, and a T*i*2-E microscope (Nikon Instruments) (Figure 2A). The following was the experimental protocol: IMCD3 cells were transfected with CMV-R-GECO using Lipofectamine 3000 Transfection Reagent (L3000015, Invitrogen) in accord with the manufacturer’s instructions, and then 80 μL of IMCD3-transfected cells at a density of 1.3 × 10^5^ cells/mL were initially seeded on a poly-l-lysine-coated glass coverslip (0111650, Paul Marienfeld, Germany) in standard cell culture medium (10% FBS), at 37 °C for 12 h. Next, serum-starvation medium (0.5% FBS) was used to replace the original medium to induce ciliogenesis. After 24 h, cells were maintained under static condition or stimulated by FUS- or VUS-induced shear stress for 30 s. The effects of FUS and VUS on IMCD3 cells were evaluated in at least four independent experiments performed under the FUS, VUS, and static conditions.

### Cilia Length and Cilia Moving Angle

The cilia of IMCD3-SSTR3-GFP cells were imaged by capturing a series of 2D fluorescence images in the *Z* direction by a T*i*2-E microscope (Nikon). The captured images were reconstructed into 3D images. Ciliary lengths of 100 cilia were assessed and analyzed using NIS-Elements software (Nikon Instruments).

The cilium deflection angle was measured in 50 cilia. The maximum cilium tip displacement was measured using NIS-Elements software, and based on the average cilia length, the cilia deflection was calculated as

$$Angle\left(\propto \right)=Arccros\left(\frac{{2h}^{2}-a^{2}}{{2h}^{2}}\right)$$

where α is the cilium deflection angle (in degrees), *h* is the average cilium length (in microns), and *a* is the average cilium tip displacement (in microns).

### Live-Cell Calcium Imaging

Calcium imaging was performed using a T*i*2-E microscope (Nikon) with a 40× air objective (Nikon). IMCD3 cells were transfected with CMV-R-GECO to assess the intracellular calcium under different experimental conditions. CMV-R-GECO-expressing IMCD3 cells were seeded onto glass coverslips at a density of 1.3 × 10^5^ cells/mL. For the cilia + static and cilia + VUS conditions, cells were cultured in the standard medium for 24 h, followed by serum-starvation medium to induce ciliogenesis. The next day, CMV-R-GECO-expressing IMCD3 cells were washed with Dulbecco’s phosphate-buffered saline (DPBS; CC704-0500, Simply) and maintained in Tyrode’s solution (PB180338, Pricella, Elabscience). Then, cells were exposed to VUS for 30 s with different parameters or maintained under static condition.

For the nonciliated + VUS condition, the serum-starvation step was not performed. After culturing on coverslips for 24 h, cells were subsequently washed with DPBS medium and replaced with new standard cell culture media at 37 °C, 24 h before being maintained in Tyrode’s solution and exposed to VUS for 30 s with different parameters.

In all experiments, cells were imaged by sequential scanning every 2 s (with a 0.5 s interval), with baseline imaging initiated 30 s before treatment under the VUS condition. Cell boundaries were manually determined for each capture. Mean cellular fluorescence intensity at each time point was quantified using ImageJ. Data are presented as mean ± SEM from 4–6 experiments per condition, with at least 70 cells analyzed per experiment.

To determine whether the VUS-induced calcium changes in IMCD3 cells depend on the primary cilia, ciliogenesis was inhibited by treatment with 5 μM Roscovitine for 24 h, as described above. Then, Fluo-4 AM (2 μM, F14201, Thermo Fisher Scientific) was used for 30 min before VUS stimulation to indicate the cellular calcium readout.

Real-time calcium fluorescence was analyzed with NIS-Elements, with time-series signals normalized across randomly assigned experiment groups and imaging fields. All experiments were independently replicated ≥ 3 times, as indicated in detail in the figure legends.

### Role of Cilia in Responses to VUS-Induced Shear Stress

The role of cilia in cellular responses to VUS-induced shear stress was investigated by culturing and treating IMCD3 cells and primary neurons under different conditions, including the cilia + static, nonciliated + VUS, and cilia + VUS conditions. On the first day, cells were placed on poly-l-lysine-coated coverslips (1 × 10^5^ and 8 × 10^5^ cells per well, respectively). After 24 h in standard growth medium, cells were maintained in either serum-starvation or control medium (IMCD3: DMEM with 0.5% FBS and 1% penicillin–streptomycin; neurons: Neurobasal added with 1× GlutaMAX, 12.5 μM l-glutamic acid, 1% penicillin–streptomycin and 0.5% B-27). Next day, cells were incubated with Fluo-4 AM for 30 min before exposed to VUS for 30 s or maintained under static conditions. All procedures were performed in the dark. Time-lapse images were acquired every 2 s for 270 s (first 30 s baseline), cell boundaries were manually defined, and mean fluorescence intensity was quantified using NIS-Elements. Data are shown as mean ± SEM from ≥ 5 independent experiments (≥250 cells per experiment).

To determine whether the calcium response to VUS-induced shear stress in IMCD3 cells and neurons depends on the presence of primary cilia, Roscovitine was used to inhibit the centriole assembly and intraflagellar transport by disrupting the key kinases Cdk2 and Cdk5 to prevent cilium formation. After seeding and culturing under standard conditions, IMCD3 cells and primary neurons were incubated in serum-starvation medium (DMEM or Neurobasal formulations as described above) supplemented with 5 μM Roscovitine (R7772, Sigma-Aldrich) in DMSO (D2650, Sigma-Aldrich) for 24 h before being incubated with Fluo-4 AM (2 μM; F14201, Thermo Fisher Scientific) for 30 min and then stimulated by VUS-induced shear stress. The same stimulation protocol was applied as in the nonciliated + VUS and cilia + VUS groups. The numbers and lengths of primary cilia were quantified in Roscovitine-treated cells to confirm the efficacy of chemical inhibition.

### Calcium Sources in the Response to VUS-Shear-Stress

The calcium sources related to VUS-induced calcium release were identified by performing two experiments. First, the Ca^2+^-specific chelator EGTA was used to bind free Ca^2+^ in the cell culture medium to block the influx of extracellular calcium. This experiment was designed to determine the contribution of extracellular calcium following stimulation by VUS-shear-stress. Second, the role of cytosolic calcium stores was determined by applying thapsigargin (a noncompetitive inhibitor of SERCA) to inhibit calcium release from internal stores. Ciliated neurons were incubated in medium with/without of EGTA (10 μM) or thapsigargin (100 nM) for 30 min in Tyrode’s solution (PB180338, Pricella, Elabscience) before adding Fluo-4 AM. The cells were then stimulated with VUS. The control group comprised ciliated neurons stimulated under the same VUS conditions without EGTA or thapsigargin treatment.

### Contribution of Ion Channels in Reply to VUS-Induced Shear Stress

To identify the contribution of ion channels in the reply to VUS-induced shear stress, GsMTx4 (2.5 μM, Ab141871, Abcam, USA), HC-067047 (1 μM, HY-100208, MCE MedChemExpress, USA), and amiloride hydrochloride (1 μM, HY-B0285A, MCE MedChemExpress) were added to the Neurobasal medium before VUS stimulation to block ciliary membrane ion channels and inhibit mechanical responses. The control group comprised neurons without the addition of blockers. Before VUS stimulation, neurons were incubated with inhibitors for 30 min. The inhibitory efficacy of ion channel blockers was recorded and analyzed based on calcium signals in 3 independent experiments (≥250 cells/experiment).

### Cell Viability Test

Cell viability was measured using the Cell Counting Kit-8 (CCK-8; CK04–01, Dojindo Laboratories) in IMCD3 cells and primary neurons cultured on poly-l-lysine-coated 25 mm coverslips. Following the cilia assembly induced by the serum-starvation medium (0.5% FBS or 0.5% B-27), cells were stimulated by VUS with different parameters (acoustic pressure = 300–500 kPa, 10–2000 cycles, PRF = 70 Hz, stimulation duration = 10–30 s). Five hours post-VUS stimulation, 10 μL of CCK-8 reagent was added per well, incubated for 2 h at 37 °C, 5% CO_2_, and absorbance was measured at 490 nm. All measurements were performed in three independent experiments. The cell viability percentage was calculated and compared with that in the untreated control group.

### Cilia Assembly Test

For the cilia assembly assay, after applying serum starvation to induce ciliogenesis (0.5% FBS), IMCD3-SSTR3-GFP cells were stimulated by VUS with different parameters (acoustic pressure = 300–500 kPa, 10–2000 cycles, PRF = 70 Hz, stimulation duration = 10–30 s). Cilia were imaged before and after VUS stimulation, and the cilia assembly ratio was calculated as the percentage of cilia present after VUS stimulation relative to the number present before stimulation. For each parameter set, samples were collected from three independent experiments, with at least nine images analyzed per condition.

### PIV Measurement

The VUS-induced flow field was measured using the PIV method. Fluoresbrite YG-labeled microspheres (17154, Polysciences, Warrington, USA) (average diameter: 1.00 μm) were dispersed (4.55 × 10^10^ particles/mL) in degassed distilled water in a metal ring, and then a VUS system was positioned on top of a glass coverslip to generate the fluid flow that would induce particle movement. A 488 nm laser line generator emitted a beam that was aligned with the xz plane. The VUS-induced flow was recorded with a CCD camera attached to an inverted microscope (Ti2-E, Nikon) at a 4× objective lens (Figure 1B). The video was processed and analyzed in MATLAB using the open-source PIVlab toolbox, with an ensemble correlation PIV algorithm adopted.

### Analysis Methods

Statistical analyses were conducted using GraphPad Prism 10, with data shown as mean ± SEM. For two-group comparisons, a two-tailed, unpaired *t*-test was applied. For multiple group comparisons, one-way or two-way ANOVA followed by Tukey’s posthoc correction was performed. *p*-value < 0.05 is demonstrated as significantly difference.

## Supplementary Material







## Notes

Figure S1. The experiment setup to evaluate the physical properties of the VUS transducer Figure S2. The experimental design for cell experiments Figure S3. The explanation of the method to measure the ciliary moving angle Figure S4. The cilia length and proportion of ciliated cells after treatment with Roscovitine Figure S5. The safety test of the VUS sonication Figure S6. Neuron viability after VUS stimulation multiple times, or without VUS stimulation (control) (PDF)
Movie S1: A primary cilium of IMCD3-SSTR-GFP cells at the upright position after single stimulation by VUS-induced shear stress (0.37 ± 0.02 dyn/cm2) for 30 s following 5 s of initial baseline recording (MP4)
Movie S2: Ciliary bending of an upright cilium in IMCD3-SSTR-GFP cells after multiple stimulation by VUS-induced shear stress (0.37 ± 0.02 dyn/cm2), with 5 s of baseline recording, followed by three 30 s stimulations with VUS-induced shear stress (0.37 ± 0.02 dyn/cm2) separated by 10 s intervals Graphic for Manuscript: The image illustrates how cilia respond to the stimulation from the VUS-induced shear stress (MP4)

## Abbreviations

VUS: Vortex Focused Ultrasound
FUS: Focused Ultrasound.
