# Supplementary material for: Modulation of Cilia Motility by Vortex-Ultrasound-Induced Shear Stress
Source: ACS Nano. 2026 Jul 15;20(29):20730–46. doi: 10.1021/acsnano.6c06231 (PMC13421962; doi:10.1021/acsnano.6c06231)
Supplement: Supplementary file 1 [file nn6c06231_si_001.pdf]

Supporting Information

Modulation of Cilia Motility by Vortex-Ultrasound-Induced Shear Stress

Thi-Nhan Phan [a], *Hsien-Chu Wang* [b], *Ching-Hsiang Fan* [c,d,e], *Chung-Han Huang* [a], *Yin Fang* [b], *Yucheng Luo* [f], *Zhichao Ma* [f], *I-Hsuan Lin* [g], *Won-Jing Wang* [g], *Yu-Chun Lin* [b,h,*], *and Chih-Kuang Yeh* [a,i,j,*]

AUTHOR INFORMATION:

Corresponding Author:

Yu-Chun Lin - Institute of Molecular Medicine, National Tsing Hua University, Hsinchu, 30013, Taiwan

Department of Medical Science, National Tsing Hua University, Hsinchu, 30013, Taiwan

ID: orcid.org/0000-0002-9629-7560; Phone: +886-3-574-2421; Email: ycl@life.nthu.edu.tw

Chih-Kuang Yeh - Department of Biomedical Engineering and Environmental Sciences, National Tsing Hua University, Hsinchu, 30013, Taiwan

Department of Biomedical Sciences and Engineering, Tzu Chi University, Hualien, 970374, Taiwan

Department of Biomedical Engineering, Chung Yuan Christian University, Taoyuan, 320314, Taiwan

ID: orcid.org/0000-0002-2880-6327; Phone: +886-3-571-5131; Email: ckyeh@mx.nthu.edu.tw; Fax: +886-3-571-8649.

Present Addresses:

a Department of Biomedical Engineering and Environmental Sciences, National Tsing Hua University, Hsinchu, 30013, Taiwan

b Institute of Molecular Medicine, National Tsing Hua University, Hsinchu, 30013, Taiwan

c Department of Biomedical Engineering, National Cheng Kung University, Tainan, 701401, Taiwan
d Center of Transformative Bioelectronic Medicine, College of Medicine, National Cheng Kung University, Tainan City, 701, Taiwan
e Medical Device Innovation Center, National Cheng Kung University, Tainan, 701401, Taiwan
f Institute of Medical Robotics, School of Biomedical Engineering, Shanghai Jiao Tong University, Shanghai, 200030, China
g Institute of Biochemistry and Molecular Biology, National Yang Ming Chiao Tung University, Taipei, 300093, Taiwan
h Department of Medical Science, National Tsing Hua University, Hsinchu, 30013, Taiwan
i Department of Biomedical Sciences and Engineering, Tzu Chi University, Hualien, 970374, Taiwan
j Department of Biomedical Engineering, Chung Yuan Christian University, Taoyuan, 320314, Taiwan

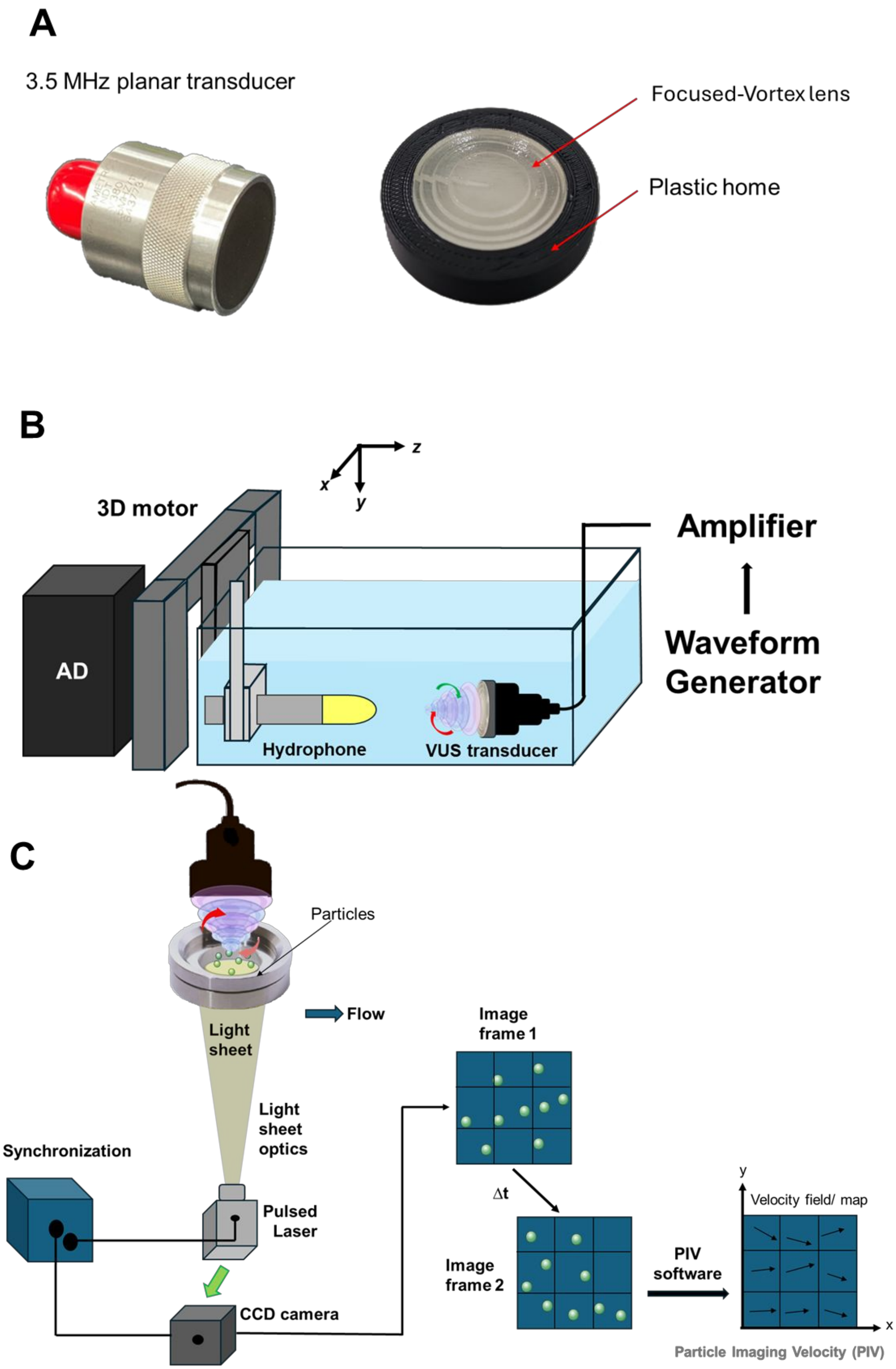
A
3.5 MHz planar transducer
Focused-Vortex lens
Plastic home
B
3D motor
AD
Hydrophone
VUS transducer
Amplifier
Waveform
Generator
z
x
y
C
Particles
Flow
Light
sheet
Light
sheet
optics
Synchronization
Pulsed
Laser
CCD camera
Image
frame 1
Δt
Image
frame 2
PIV
software
Velocity field/ map
y
x
Particle Imaging Velocity (PIV)

Figure S1. The experiment setup to evaluate the physical properties of the VUS transducer. (A) The photos of the 3.5-MHz planar transducer and the focused-vortex lens that are used to fabricate the VUS transducer. (B) The scheme of the hydrophone system to calibrate the VUS transducer. The acoustic field and energy of a 3.5-MHz planar transducer (30 mm diameter) combined with a focused-vortex lens were calibrated by a 3D automatically controlled system and a hydrophone. (C) The scheme for particle image velocimetry measurements with real-time microscopic observation. The movement of 1 μm YG-labeled microspheres was captured by a Nikon fluorescence microscope at a frame rate of 50 frames per second. The timeline for the PIV experiment was 10 s before exposure to VUS as the baseline, followed by 30 s of VUS stimulation, and 20 s post-VUS stimulation.

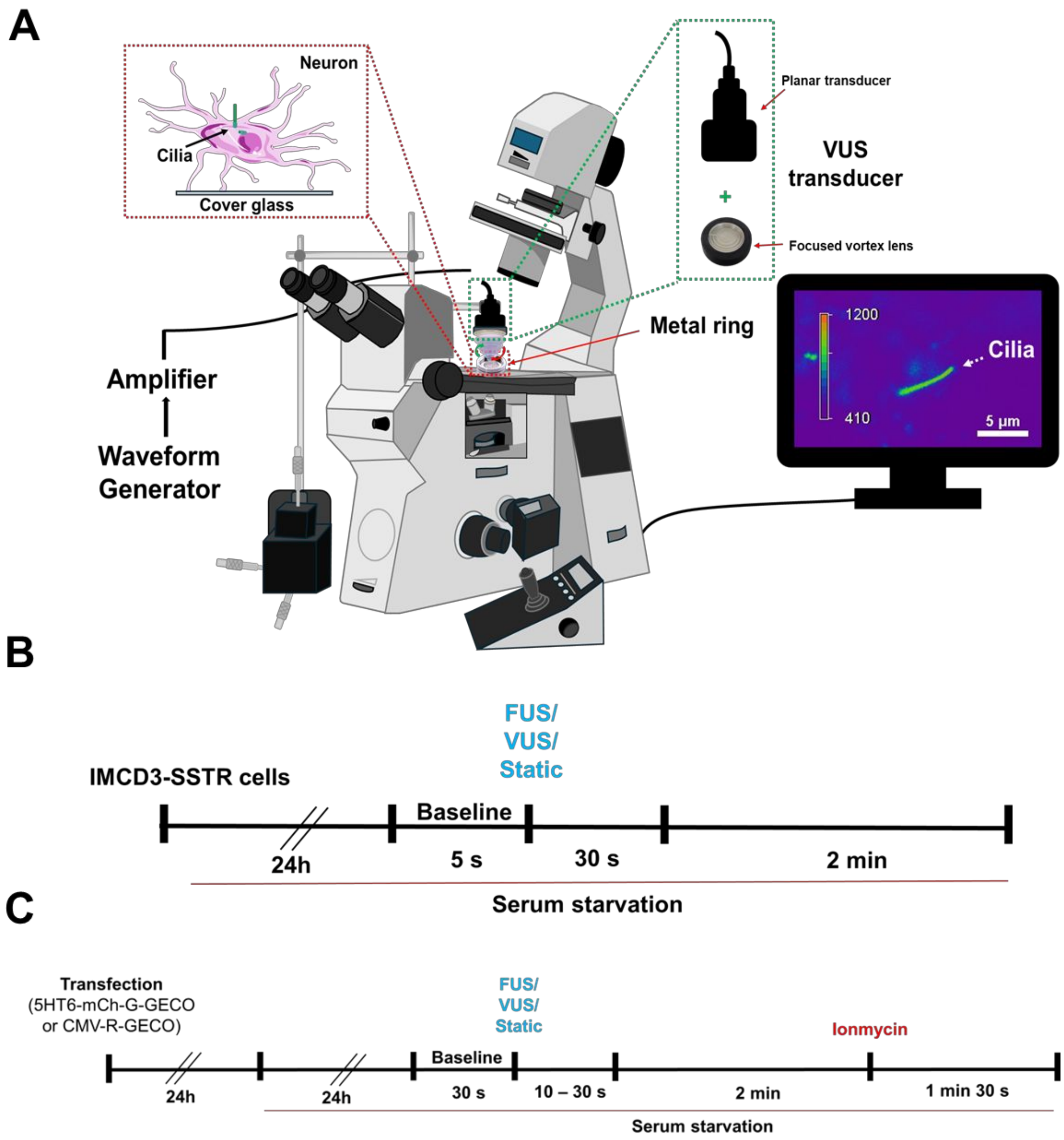

Figure S2. The experiment design for cell experiments. (A) The experimental design for VUS-mediated ciliary movement and intraciliary and intracellular calcium influxes in IMCD3 cells and neurons, with real-time microscopic observation. The water cone of the VUS transducer was immersed in the metal ring with cell-cultured cover glass and Tyrode's solution. (B) The timeline of stimulating the cilia motility under different stimulation conditions (static, FUS, and VUS). (C) The flowchart of the VUS shear stress induced the intraciliary calcium influx in IMCD3 cells. IMCD3 cells were transfected with 5HT6-mCh-G-GECO or CMV-R-GECO and seeded onto

poly-L-lysine-coated cover glass, then cultured in normal medium (DMEM + 10% FBS). After 24h, the old medium was sucked out and replaced by a serum starvation medium (DMEM + 0.5% FBS) and cultured for 24h to induce the ciliary assembly before the following experiment. On the third day, the cells were placed on a metal ring filled with commercial Tyrode's solution and then put on the VUS-fluorescence microscope system. The images were captured 30 s before treatment as the baseline. Then, the cells were treated to different stimulation conditions (static, FUS, and VUS). The ciliary calcium influx was observed for 2 min after FUS/ VUS exposure. The control calcium indicator (Ionmycin, 10 μL) was added 2 min after post-treatment.

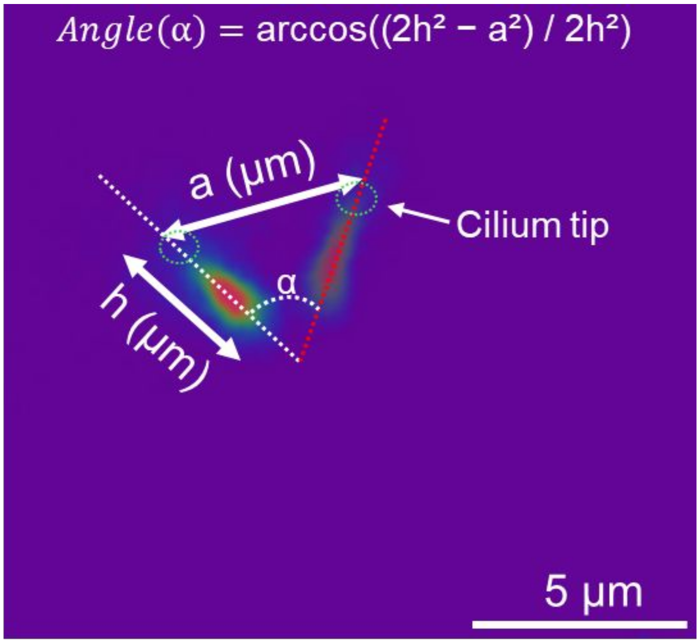

Figure S3. The explanation of the method to measure the ciliary moving angle. The average cilia length was measured using z-stack images captured from 50 cilia. The movement distance of cilia was defined by the movement of the cilia tip through the NIS-Elements AR software of Nikon. The average cilium tip displacement distance (a μm) was calculated from the baseline position to the largest position that cilia could move. From the moving distance (a μm) and the average cilia length (h μm), the average cilium moving angle was calculated.

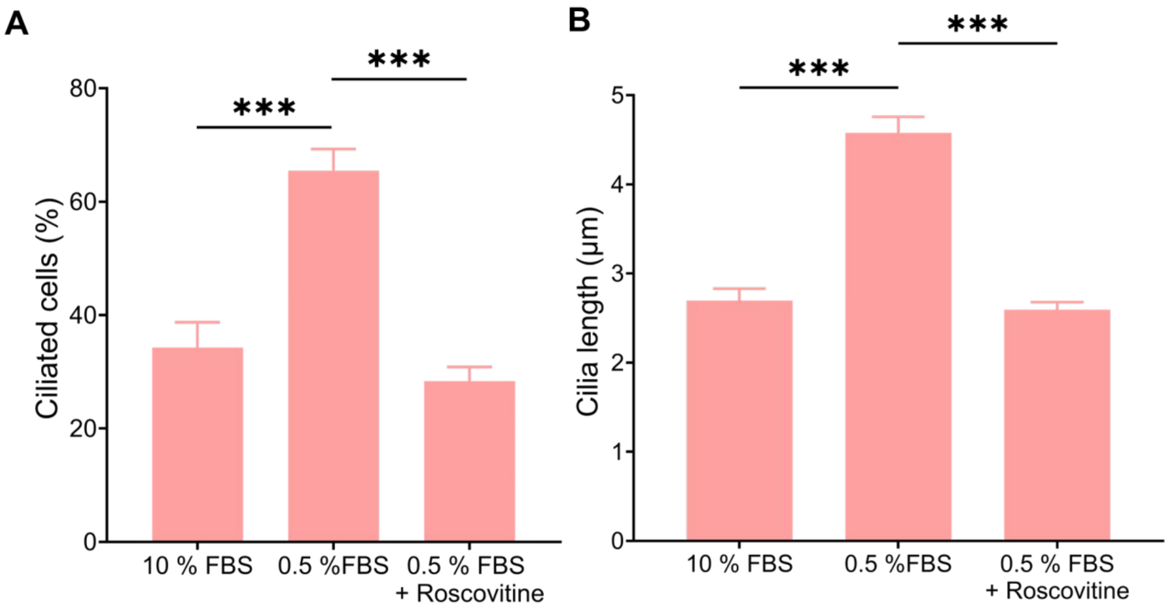

Figure S4. The cilia length and proportion of ciliated cells after treating with Roscovitine. Under serum-starvation conditions (0.5 % FBS), inner medullary collecting duct-3 (IMCD3) cells transfected with the somatostatin receptor 3 (SSTR3) structure extend primary cilia that are SSTR3-positive (green); the nuclei of IMCD3 cells were indicated by DAPI (blue). After treating with Roscovitine medium (5 μM), both the proportion of ciliated cells (A) and cilia length (B) reduced significantly compared to the control group (DMEM + 10% FBS) or without treating with Roscovitine (n = 100, with three independent experiments).

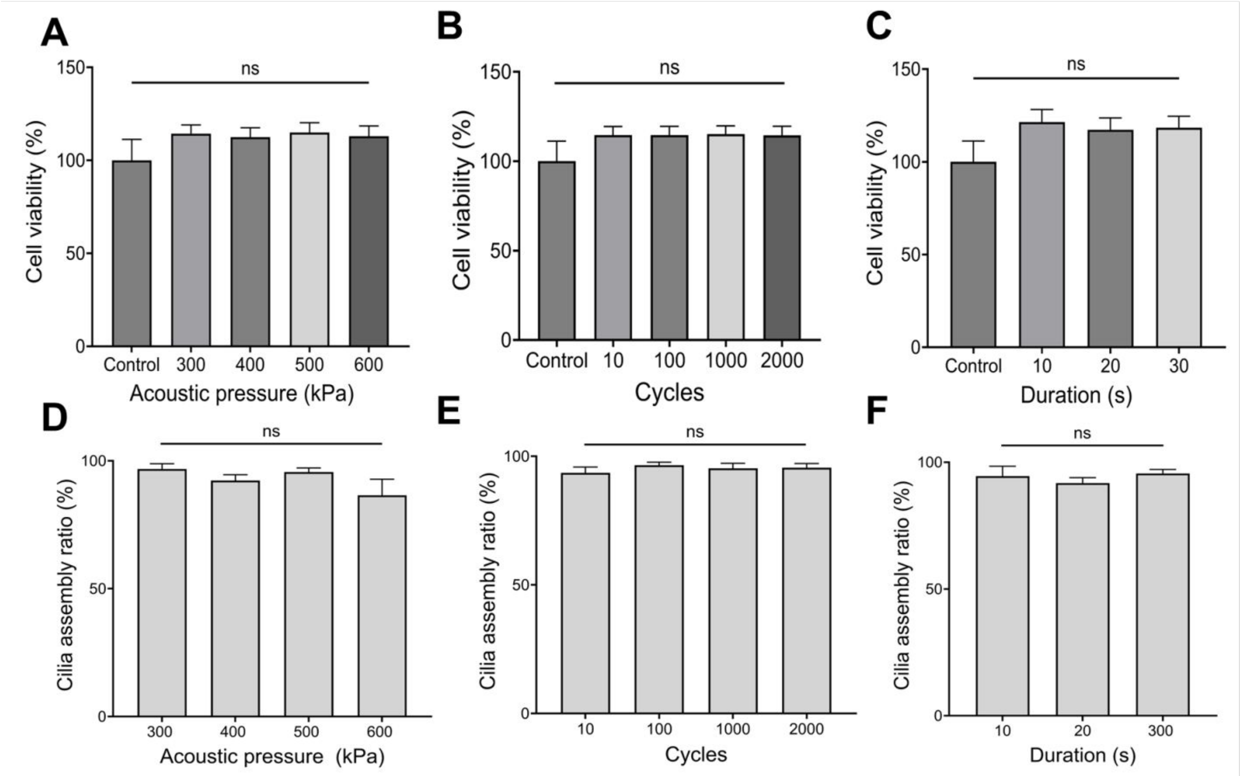

Figure S5: The safety test of the VUS sonication. (A - C) The cell viability of IMCD3 cells after being stimulated by different VUS parameters. (D - F) The cilia assembly ratio of IMCD3-SSTR cells after being stimulated by different VUS parameters. Data on cell viability are expressed as mean ± SEM (n = 18 replications, from three independent experiments) (ns: not significant, *: $p < 0.05$).

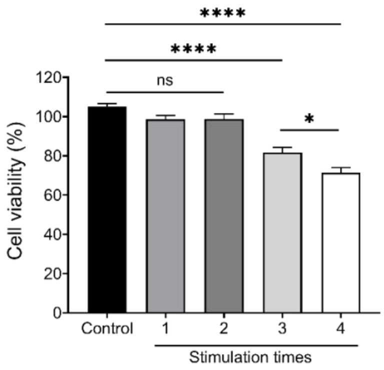

Figure S6. Neuron viability after VUS stimulation multiple times, or without VUS stimulation (control). Data on cell viability are expressed as mean ± SEM (n = 250 cells, from at least three independent experiments, ns: not significant, *: $p < 0.05$, ****: $p < 0.001$).
